# The Effect Mechanism of N6-adenosine Methylation (m6A) in Melatonin Regulated LPS-induced Colon Inflammation

**DOI:** 10.7150/ijbs.95316

**Published:** 2024-04-15

**Authors:** Yuanyuan Li, Baochen Ma, Zixu Wang, Yaoxing Chen, Yulan Dong

**Affiliations:** 1 Laboratory of Neurobiology, College of Veterinary Medicine, China Agricultural University, Beijing, People's Republic of China.; 2 Key Laboratory of Precision Nutrition and Food Quality, Ministry of Education, Department of Nutrition and Health, China Agricultural University, Beijing, People's Republic of China.

**Keywords:** Melatonin, Inflammation, Mettl3, Gut Microbiota, Macrophage

## Abstract

Colon inflammation is characterized by disturbances in the intestinal microbiota and inflammation. Melatonin (Mel) can improve colon inflammation. However, the underlying mechanism remains unclear. Recent studies suggest that m6A methylation modification may play an important role in inflammatory responses. This study aimed to explore the effects of melatonin and LPS-mediated m6A methylation on colon inflammation. Our study found that melatonin inhibits M1 macrophages, activates M2 macrophages, inhibit the secretion of pro-inflammatory factors, maintain colon homeostasis and improves colon inflammation through MTNR1B. In addition, the increased methylation level of m6A is associated with the occurrence of colon inflammation, and melatonin can also reduce the level of colon methylation to improve colon inflammation. Among them, the main methylated protein METTL3 can be inhibited by melatonin through MTNR1B. In a word, melatonin regulates m6A methylation by improving abnormal METTL3 protein level to reshape the microflora and activate macrophages to improve colon inflammation, mainly through MTNR1B.

## Introduction

Intestinal inflammation is a condition caused by an abnormal immune system reaction. Inflammatory bowel diseases (IBD) 1, irritable bowel syndrome (IBS) 2, and intestinal ischemia-reperfusion injury 3, the pathogenesis of these diseases remains poorly understood, but they are all characterized by repetitive and chronic inflammatory changes in the gut. Excessive inflammatory hyperplasia also induces intestinal cancer 4,5. Most studies suggest that the occurrence of intestinal inflammation can be attributed to imbalances in the gut microbiota, immune system abnormalities, and environmental factors, et al 6-9. Therefore, reducing intestinal inflammation is very important and can become an effective method of treatment for intestinal-inflammation-related digestive diseases 10.

Melatonin (Mel) is the major product of the pineal gland and has a strong anti-inflammatory capacity 11. As the first line of defense in innate immunity, macrophages can be activated by LPS to differentiate into M1 macrophages, secreting chemokines and inflammatory cytokines to participate in regulating inflammation in the body 12,13. It was indicated melatonin suppressed the production of nitric oxide (NO) and interleukin-6 (IL-6) at both gene transcription and translation levels in lipopolysaccharide (LPS)-activated macrophages 14. Therefore, melatonin may have a certain role in improving intestinal inflammation, but its specific mechanism of action still needs further exploration.

N6-methyladenosine (m6A) is methylation that occurs in the N6-position of adenosine, which is the most abundant mRNA modification 15-17. Similar to epigenetic changes in DNA and histone modifications, m6A in RNA is dynamic and reversible 18,19. The m6A methylation is catalyzed by the methyltransferase complex including METTL3, METTL14, and WTAP, whereas its demethylation is mediated by FTO and ALKBH5 20,21. A recent study demonstrated that METTL3-mediated m6A methylation promotes dendritic cell activation 22. Viral infection can increase the levels of host m6A modification, which negatively regulates the type I interferon response 23. In addition, m6A modification can regulate inflammation and apoptosis, which are important mechanisms mediating pathological injury in inflammation. However, the role of m6A in colon inflammation response is still poorly understood, the molecular mechanism of m6A for melatonin-mediated anti-inflammatory role was unknown.

Here we show that melatonin inhibited effects LPS-induced m6A methylation level up-regulated and protected the colon barrier. Importantly, inhibition of mettl3, an important protein in m6A methylation process, changes the type of macrophages and protects the colon barrier, while inhibition of MTNR1B eliminates the effect of melatonin. We suggest that LPS-induced inflammation and changes in macrophage may treat inflammation by inhibiting m6A methylation levels, and that melatonin regulates m6A methylation levels through MTNR1B, providing a new insight into the role of melatonin.

## Materials and Methods

### Animals and treatment

**Figure 1:**
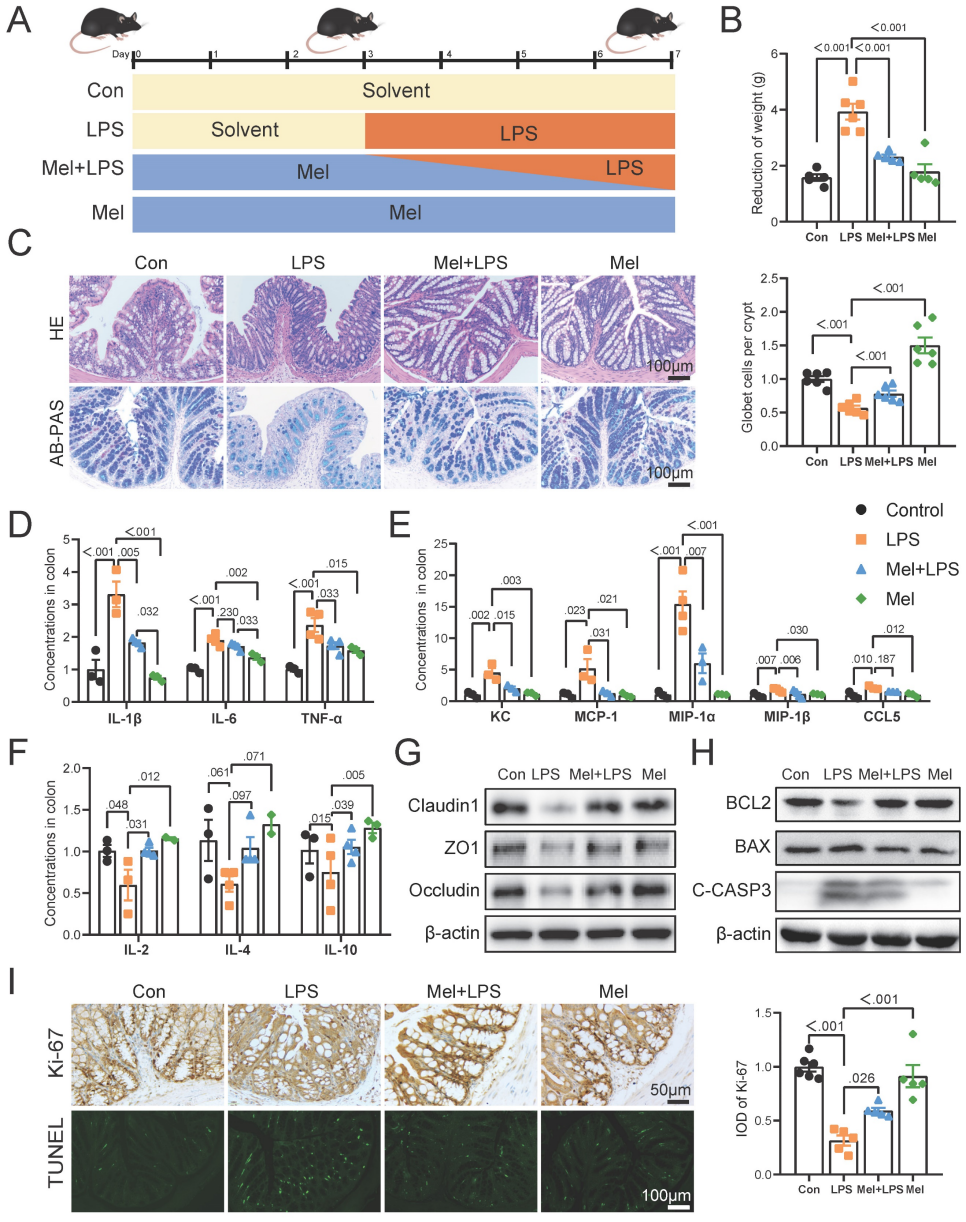
Melatonin improved colon inflammation induced by LPS. (A) Illustration of the experimental design. (B) The reduction weight (n = 6). (C) H&E and AB-PAS staining and quantification of globet cells per crypt (n = 6). (D-F) Pro-inflammatory factors (D), chemokines (E) and anti-inflammatory factors (F) protein levels (n = 3-4). (G) Claudin1, ZO1 and Occludin protein expression. (H) BCL2, BAX and C-CASP3 protein expression. (I) Ki-67 and TUNEL staining and IOD of Ki-67 (n = 5-6). Data are expressed as the mean ± SEM. One-way ANOVA. All p values are shown in the figures.

A total of 90 female C57BL/6J mice were purchased from Beijing Vital River Laboratory Animal Technology Co., Ltd. The mice were treated with LPS and Melatonin. LPS (L2880, Sigma) was prepared fresh daily in 0.9% saline and melatonin (M5250, Sigma) was prepared fresh daily in 3% ethanol, stored at 4 °C. Experiment 1: Animals were divided into the following groups: Control (3% ethanol+saline), LPS (3% ethanol+LPS), LPS+Mel (Mel+LPS), Mel (Mel+saline). LPS (5 mg/kg), Mel (20 mg/kg), ethanol and saline was injected i.p. (Fig. 1A). Experiment 2: Animals were divided into the following groups: Control (3% ethanol+saline), LPS (3% ethanol+LPS), Mel+LPS (Mel+LPS), SAH+LPS (SAH+Mel), 4-P-PDOT+Mel+LPS (4-P-PDOT+Mel+LPS). SAH (8 mg/kg), 4-P-PDOT (1 mg/kg), ethanol and saline were injected i.p. (Fig. 7A). The tissue was stored at -80°C for experiment. The colon content was put into the sterilized cryopreservation tube, and stored in liquid nitrogen for microbial analysis. Portions of the colon were placed in 4% paraformaldehyde for histological and immunohistochemical observation. All animal procedures were approved by the China Agricultural University Institutional Animal Care and Use Committee (AW03602202-2-1).

### H&E and AB-PAS staining

Colon tissues were embedded in paraffin, and serial 5-μm-thick sections were placed onto slides. After dewaxing and rehydration, the sections were stained with H&E according to routine protocols. For AB-PAS staining, colon sections were stained using the Alcian Blue Periodic Acid Schiff (AB-PAS) Stain Kit (G1285, Solarbio).

### Immunohistochemistry staining

After deparaffinization and rehydration, colon sections were soaked in sodium citrate buffer for heat-induced epitope exposure. Nonspecific binding sites were blocked by incubating with 10% goat serum at 37°C for 1 h. Then, the sections were incubated overnight with in primary antibody overnight at 4°C. Followed by incubation with biotinylated goat anti-rabbit IgG secondary antibodies (A0277, Beyotime) for 2 h. After treatment with diaminobenzidine (DAB) Kit (PV-6001, ZSGB Biotech Co., Inc.) and counterstained with hematoxylin. All images were taken using an Aperio CS2 scanner (Leica Biosystems, Netherlands). Quantification of IHC images was performed using Image J software (Media Cybernetics Inc., Silver Springs, MD). The primary antibodies used were as follows: METTL3 (15073-1-AP, Proteintech), Ki-67 (ab15580, Abcam).

### Immunofluorescence staining

Colon sections were selected after deparaffinization was performed with 10 mM, pH 6.0 sodium citrate solution in 95°C water bath for 10 min, followed by a 20 min incubation at room temperature. Slides were washed, blocked in 5% normal goat serum at 37°C, and stained using the primary antibodies that rabbit anti-F4/80 antibody (29414-1-AP, Proteintech) and mouse anti-CD206 antibody (60143-1-Ig, Proteintech) overnight at 4°C, respectively. Then sections were incubated with goat anti-rabbit alexa fluor 594 (ab150080, Abcam) and goat anti-mouse alexa fluor 488 (A0428, Beyotime) for 1 hour. The sections were photographed with a confocal microscope (FLUOVIEW FV3000, Olympus) and analyzed with Image J software (National Institutes of Health, Bethesda, MD).

Cell samples were fixed for 30 min by 4% paraformaldehyde, permeated for 10 min in 0.03% Triton-X-100. Then, 5% goat serum was incubated for 30 min at 37°C, and incubated in METTL3 antibody (15073-1-AP, Proteintech) overnight at 4°C. The next day, cells were incubated with goat anti-rabbit alexa fluor 594 (ab150080, Abcam) for 1 hour, DAPI stained the nuclei for 10 min, and then photographed.

### TUNEL staining

For TUNEL staining, colon sections were treated with a TUNEL kit (B0013, LABLFAD) according to the manufacturer's instructions. TUNEL-positive cells were observed under Nikon Eclipse TE 2000S inverted microscope (Nikon Instruments, Inc).

### RNA extraction and qRT-PCR

Total RNA was isolated with TRIzon Reagent (R401-01, Vazyme). The concentration and purity of RNA was measured with a Nanophotometer (Implen, Munich, Germany). Then, cDNA was synthesized with HiScript QRT supermix for qPCR (Q142, Vazyme). Real-time quantitative PCR (qPCR) was performed with SYBR green master mix (Q142, Vazyme). Changes in fluorescence were monitored on a OneStep Plus instrument (Applied Biosystems). The primer sequences used for qPCR were as shown in Table 1.

### LC-MS/MS

Total m6A was measured in 1 μg of total RNA extracted using liquid chromatography-tandem mass spectrometry (LC-MS/MS). RNA was incubated with 1 μL of S1 nuclease (2410A, Takara) for 4 hours at 37°C. Then, 1 μL of alkaline phosphatase (2660A, Takara) was added, and the reaction was incubated for 1 hour at 37°C. The reaction mixture was extracted with chloroform. HPLC separation was performed using a C18 column (Shimadzu Corporation) with a flow rate of 0.2 mL/min at 35 °C. Solvent A was 0.1 % (vol/vol) formic acid in water, and solvent B was 0.1 % (vol/vol) formic acid in methanol. A gradient of 5 min of 5 % B, 10 min of 5-30% B, 5 min of 30-50% B, 3 min of 50- 5% B, and 17 min of 5 % B was used.

### Luminex liquid suspension chip detection

Luminex liquid suspension chip detection was performed by Wayen Biotechnologies (Shanghai, China). The Bio-Plex Pro Human Chemokine Panel 23-plex kit was used in accordance with the manufacturer's instructions. In brief, the protein concentrations of colon were determined using a bicinchoninic acid (BCA) (μg/mL) (CW0014S, Cwbio). Then colon proteins incubated with detection antibody for 30 min. Subsequently, streptavidin-PE was added into each well for 10 min, and values were read using the Bio-Plex MAGPIX System (Bio-Rad).

### Western blot

The colon was rapidly isolated and lysed in RIPA lysis buffer (P0013B, Beyotime) containing 1% protease inhibitor cocktail (539131, Solarbio) and 1% phosphatase inhibitor cocktail (P1260, Solarbio). The lysates were centrifuged at 12,000 rpm for 15 min at 4 °C. The supernatants were collected, and the amount of protein was measured using a bicinchoninic acid (BCA) (CW0014S, Cwbio), before the protein concentration was standardized. The protein samples were resolved using 10%-15% sodium dodecyl sulfate-polyacrylamide gel electrophoresis (SDS-PAGE) and electro blotted onto a polyvinylidene fluoride membrane (Millipore, Billerica, MA). Nitrocellulose membranes were blocked for 1 hour using TBST (Tris-buffered saline solution with Tween 0.1%) containing 5% fat-free dry milk. They were then incubated in primary antibodies overnight at 4 °C. After washing with TBST, they were incubated with the secondary antibody anti-rabbit/mouse IgG DyLight conjugate (SE134, Solarbio) for 2 hours at room temperature, and imaged with a Sapphire Biomolecular Imager (Azure Biosystems). The protein band intensities were quantified using ImageJ software (Nation-al Institutes of Health, Bethesda, MD, USA).

The primary antibodies used were as follows: METTL3 (15073-1-AP, Proteintech), FTO (27226-1-AP, Proteintech), PARP (T40050S, Abmart), Cleaved caspase-3 (19677-1-AP, Proteintech), BAX (50599-2-Ig, Proteintech), BCL2 (68103-1-Ig, Proteintech), Occludin (27260-1-AP, Proteintech), Claudin-1, (ab211737, Abcam), ZO1 (21773-1-AP, Proteintech), TLR4, (sc-293072, santa), p-P65 (ab31624, Abcam), p-IκB (ab31625, Abcam), CD206, (TU313804S, Abmart), CD68, (28058-1-AP, Proteintech), iNOS, (TA0199S, Abmart), β-actin (66009-1-Ig, Abcam).

### Microbial sequencing

The microflora detection was completed by Baimaike Company. The first step was to obtain bacterial genomic DNA from frozen colon contents. DNA samples were PCR amplified by using bar-coded primers flanking the V3-V4 region of the 16S rRNA gene. High-throughput pyrosequencing of the PCR products was then performed on the company's Illumina MiSeq 2500.

### Data set

We collected tissue-specific mRNA expression data from public datasets (GSE47908 with 15 normal people and 39 IBD patients were analyzed from colonic mucosal biopsy). The EdgeR package (edgeR 3.14.0) and limma package (version 3.30.7) were used to identify differentially expressed genes (DEGs) between the Con and IBD groups (p-value < 0.05 and fold change > 2).

### Cell culture

RAW264.7 and Caco2 cells were cultured in DMEM with 100 units/mL penicillin, 100 mg/mL streptomycin, and 10% fetal bovine serum (FBS) (EVERY GREEN, 70220-8611) in a CO_2_ incubator (5% CO_2_, 37 °C). The RAW264.7 cells were stimulated with LPS (1 ug/ml) and melatonin (1 µm) for 12 hours. Caco2 cells were stimulated with LPS (50 ug/ml) and melatonin (2 µm) for 24 hours. For inhibitors, cells were incubated with 1 μM SAH (HY-19528, MCE) and 100 nM 4-P-PDOT (HY-100609, MCE) for 24 hours. Cells were washed three times by ice-cold PBS buffer to remove the cell culture medium. Samples were collected for subsequent experiments.

### Flow cytometric analyses

The cell apoptosis was detected by flow cytometric analyses to investigate. Briefly, after drug treatment, Caco2 cells were digested with 0.25% trypsin, and the lysates were centrifuged at 3000 rpm for 5 min, washed once with PBS. Then, cells were incubated with the Annexin V-FITC and a propidium iodide (PI) solution (HY-K1073, MCE) for 15 min at room temperature away from light. The percentage of apoptotic cells for each sample was subsequently evaluated by a BD FACSCalibur flow cytometer (BD Biosciences, Franklin Lakes).

### Statistical analysis

Statistical analysis was performed with GraphPad Prism 8.01. One-way analysis of variance (ANOVA) was used for measurement data of more than two groups. All general statistical analysis was calculated with a confidence interval of 95%. P values ≤ 0.05 were considered as statistically significant (*p < 0.05, **p< 0.01, ***p < 0.001). Data are represented as means ± SEM as indicated in the figures.

## Results

### Melatonin reversed inflammation induced by LPS

Inflammation was modeled by intraperitoneal injection of LPS (Fig. 1A). Firstly, melatonin increased the weight induced by LPS (Fig. 1B). H&E staining showed that melatonin decreased LPS-induced inflammatory infiltration in the colon (Fig. 1C). AB-PAS staining showed that the number of goblet cells were significantly decreased after LPS treatment, which was improved by melatonin (Fig. 1C). The pro-inflammatory factors (IL-1β, IL-6, and TNF-α) (Fig. 1D) and chemokines (KC, MCP-1, MIP-1α, MIP-1β, and CCL5) (Fig. 1E) protein levels were significantly increased after LPS treatment, while the anti-inflammatory factors (IL-2, IL-4 and IL-10) were significantly decreased, and melatonin reversed LPS-induced trend (Fig. 1F). As showed in Fig.1G, melatonin restored Claudin1, ZO1 and Occludin protein level decreased by LPS (Fig. 1G). Then, melatonin inhibited BAX and C-Casp3 and increased BCL2 protein expression which was changed by LPS (Fig. 1H). Finally, Ki-67 staining which showed the proliferation ability was decreased by LPS, while melatonin improved the proliferation ability inhibited by LPS (Fig. 1I). On the contrary, TUNEL staining which showed the apoptosis level has opposite trend (Fig. 1I). These results collectively indicated that melatonin alleviated LPS-induced colon tight junction protein reduction, cell apoptosis and improved inflammation.

### Effect of LPS and melatonin on colon microbiota

**Figure 2:**
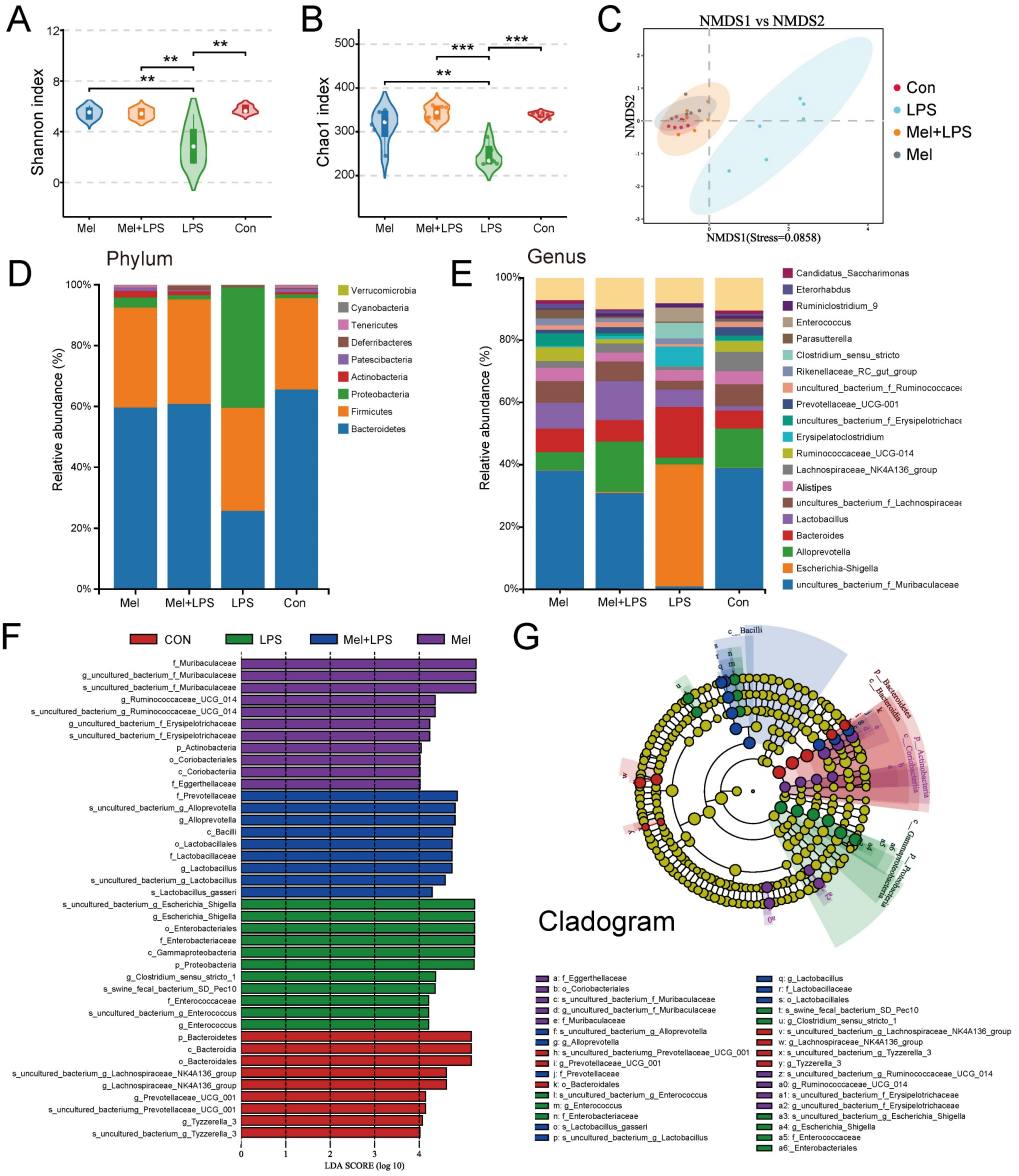
Composition of the colon microbiota after LPS and melatonin treatment. (A-B) Shannon (A) and Chao1 (B) diversity indexes. (C) NMDS score plots based on the OTUs (n = 6). (D) Relative abundance of the top 10 genera at the phylum levels. (E) Relative abundance of the top 20 genera at the genus levels. (F-G) LEfSe analysis (Cladogram and LDA score) (LDA score > 4.0). *P < 0.05, ** P < 0.01 *** P < 0.01.

Significant differences were observed in both alpha and beta diversity as well as in specific relative abundancies in four groups. The species diversities of microbiome samples (alpha diversity) were estimated by chao1 index and shannon index (Fig. 2A, B), which was decreased in LPS group (P<0.05, vs Con group), but profoundly restored after melatonin treatment (P<0.01, vs LPS group). The diversities between microbiome samples (beta diversity), as determined by the non-metric dimensional scaling (NMDS), showed that cluster of LPS group was relatively separated (P<0.01, vs other groups), suggesting the distinguished bacterial structures among groups (Fig. 2C). A histogram of species distribution can intuitively reflect changes in fecal microbiota composition. At the phylum levels, Bacteroidetes, Firmicutes, and Proteobacteria were main flora (Fig. 2D). At the genus levels, uncultured_bacterium_f_Muribaculaceae, Escherichia-Shigella, Alloprevotella, and Bacteroides were predominant (Fig. 2E). LEfSe analysis was used to compare the microbial composition and specific bacterial taxa in each experimental group ('c_', class, 'o_', order, 'f_', family, 'g', genus, 'p', phylum). The results showed the LDA fraction of these bacteria, indicating predominant bacteria in four groups (Fig. 2F, G).

### LPS and melatonin affected m6A methylation levels in the colon

**Figure 3:**
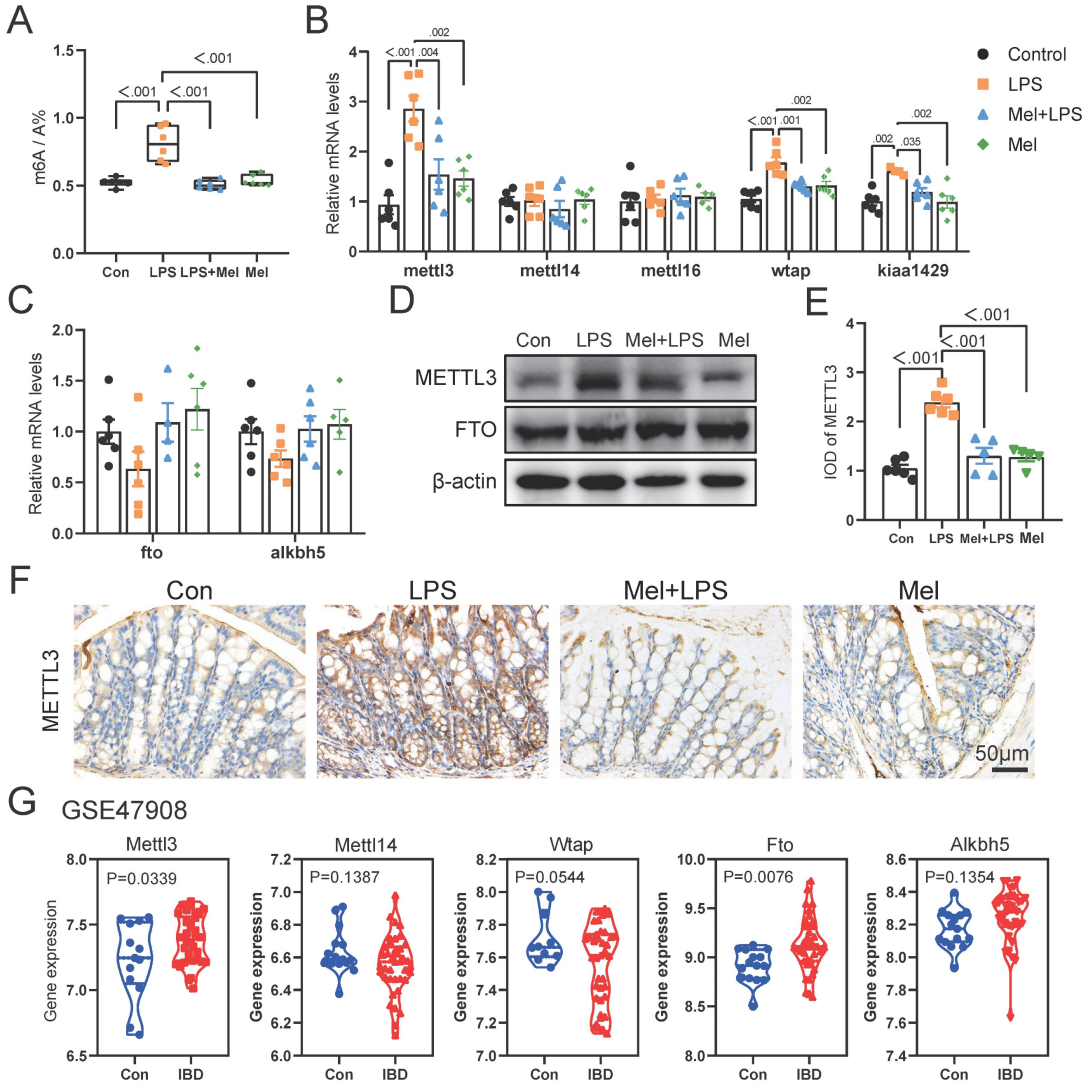
Changes of m6A methylation-related genes in colon. (A) m6A levels of colon by LC-MS (n = 6). (B) The m6A methylase mettl3, mettl14, mettl16, wtap and kiaa1429 mRNA levels (n = 6). (C) The m6A demethylase fto and alkbh5 mRNA levels (n = 5-6). (D) METTL3 and FTO relative protein expression (n = 6). (E) IOD of METTL3 (n = 5-6). (F) METTL3 staining. (G) The expression level of Mettl3, Mettl14, Wtap, Fto and Alkbh5 between the Con and IBD. Data are expressed as the mean ± SEM. One-way ANOVA. All p values are shown in the figures.

We first examined m6A methylation levels in colon and found that melatonin could reduce the abnormal increase in m6A methylation levels induced by LPS (Fig. 3A). The mRNA levels of mettl3, wtap and kiaa1429 was increased after LPS treatment, and melatonin inhibited its mRNA levels that LPS-increased (Fig. 3B). The mettl14, mettl16, fto and alkbh5 mRNA levels had no significant change (Fig. 3C). METTL3 staining showed that METTL3 was mainly widely expressed in colon cells, and the expression of METTL3 was significantly increased by LPS, while melatonin reduced the expression of METTL3 (Fig. 3E, F). METTL3 protein expression were consistent with the mRNA trends, and FTO had no significant change (Fig. 3D). We downloaded transcriptome sequencing data of colonics mucosal biopsy from 15 normal people (Con) and 39 patients (IBD) with colitis from the GEO database (GSE47908). We analyzed the mRNA levels of m6A regulators from the dataset. We found that the m6A writers Mettl3, Wtap were obviously higher and Kiaa1429 was lower in IBD group (Fig. 3G). The mRNA levels of erasers Fto was higher in IBD group (Fig. 3G). These results suggested that m6A regulators METTL3 which were abnormally regulated may involve the development of colon inflammation.

### LPS worked through TLR4/NF-κB pathway of macrophages and melatonin worked through MTNR1B in colon

**Figure 4:**
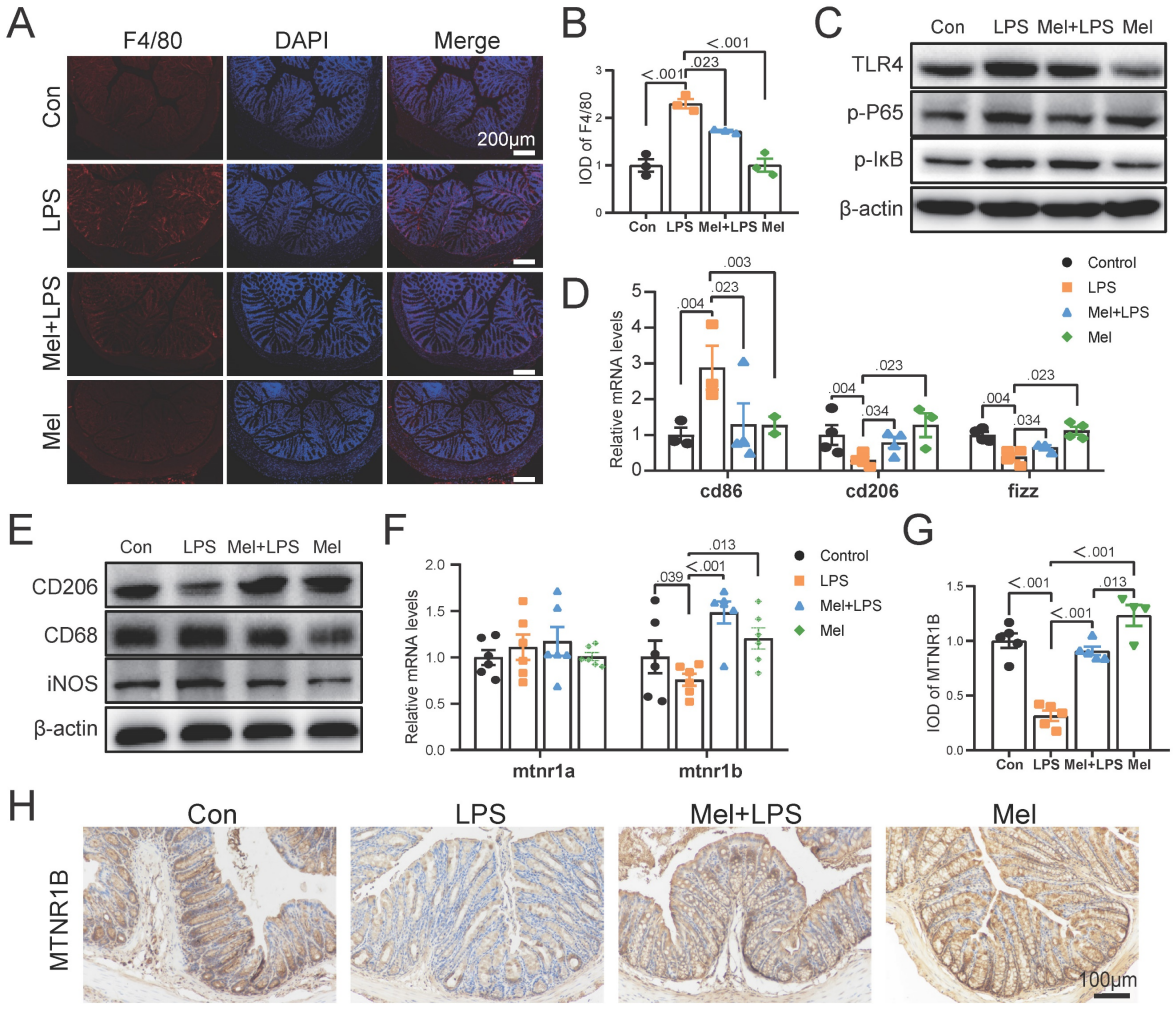
LPS worked through TLR4/NF-κB pathway of macrophages and melatonin worked through MTNR1B. (A) F4/80 staining. (B) IOD of F4/80 (n = 3). (C) TLR4, p-P65 and p-IKB protein expression. (D) cd86, cd206 and fizz mRNA levels (n = 3-4). (E) CD206, CD68 and iNOS protein expression. (F) mtnr1a and mtnr1b mRNA levels (n = 6). (G) IOD of MTNR1B (n = 4-5). (H) MTNR1B staining. Data are expressed as the mean ± SEM. One-way ANOVA. All p values are shown in the figures.

As the first line of defense of innate immunity, macrophages play a key role in the occurrence, development and elimination of inflammation. The immunofluorescence of F4/80 showed that LPS increased the expression of macrophage markers, while melatonin decreased it (Fig. 4A, B). Through western blotting detection, it was showed that LPS activated the NF-κB pathway in the colon. The expression of TLR4, p-P65 and p-IκB proteins were increased compared to the Con group, and melatonin decreased TLR4, p-P65and p-IκB protein expression compared to the LPS group (Fig. 4C). We detected the mRNA levels of macrophages markers, and found that LPS increased M1 macrophages marker (cd86) mRNA levels and decreased M2 macrophages marker (cd206 and fizz) mRNA levels compared to the Con group, melatonin effectively improved the levels change caused by LPS (Fig. 4D). Then we detected the protein expression of macrophage markers (CD206, CD68 and iNOS) (Fig. 4E). LPS increased the expression of CD68 and iNOS proteins and decreased the expression of CD206 protein, whereas melatonin inhibited these effects.

To further investigated how melatonin worked, we detected the mRNA levels of mtna1a and mtnr1b and found melatonin may worked through MTNR1B because of significant differences compared Mel+LPS group with LPS group (Fig. 4F). Moreover, the immunohistochemical staining of MTNR1B also proved it (Fig. 4G-H). These results indicated that LPS induces colon inflammation through TLR4/NF-κB pathway, mainly promoting the increase of M1 macrophages, while melatonin promotes M2 macrophages through MTNR1B receptors.

### SAH inhibited the inflammatory response of macrophages and 4-P-PDOT promoted the effect of LPS

**Figure 5:**
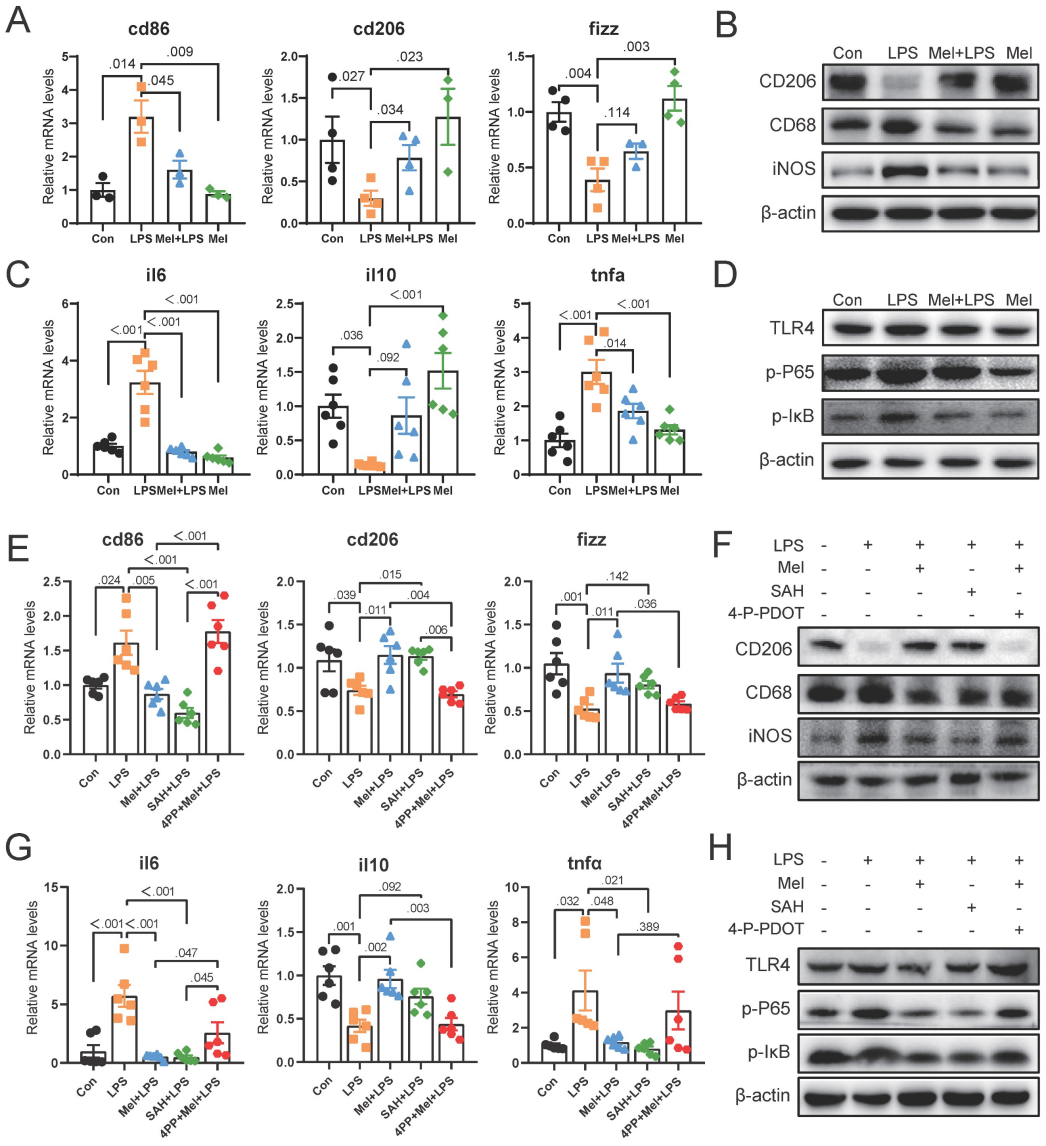
SAH inhibited the inflammatory response of Macrophages and 4-P-PDOT promoted the effect of LPS. (A) cd86, cd206 and fizz mRNA levels (n = 6). (B) CD206, CD68 and iNOS protein expression. (C) il6, il10 and tnfα mRNA levels (n = 6). (D) TLR4, p-P65, and p-IκB protein expression. (E) Macrophage marker cd86, cd206 and fizz mRNA levels (n = 6). (F) CD206, CD68 and iNOS protein expression. (G) il6, il10 and tnfα mRNA levels (n = 6). (H) TLR4, p-P65, and p-IκB protein expression. Data are expressed as the mean ± SEM. One-way ANOVA. All p values are shown in the figures.

To further determine the potential molecular mechanism for m6A effect in LPS and melatonin treatment. We examined the mRNA levels of cd206 and fizz, which were restored by melatonin but inhibited by LPS. Conversely, LPS increased cd86 mRNA levels and melatonin decreased its (Fig. 5A). Similarly, LPS decreased the protein expression of CD206 and increased the protein expression of CD68 and iNOS, melatonin inhibited the effects of LPS on CD68, iNOS, and CD206 protein expression (Fig. 5B). Additionally, LPS increased il6 and tnfα mRNA levels, inhibited il10 mRNA levels, and melatonin improved its (Fig. 5C). Furthermore, melatonin decreased TLR4, p-P65, and p-IκB protein expression, which was increased by LPS (Fig. 5D).

Subsequently, we treated RAW264.7 cells with SAH (an inhibitor targeting the METTL3-METTL14 heterodimer complex) and 4-P-PDOT (melatonin receptor MTNR1B antagonist). We observed that SAH significantly decreased cd86 mRNA levels and CD68 and iNOS protein expression, while increasing cd206 and fizz mRNA levels and CD206 protein expression compared to the LPS group (Fig. 5E, F). Additionally, SAH reduced il6 and tnfα mRNA levels, while enhancing il10 mRNA levels compared to the LPS group (Fig. 5G). Furthermore, SAH inhibited the overexpression of TLR4, p-P65, and p-IκB protein expression compared to the LPS group (Fig. 5H). These results further support the effective inhibition of LPS-induced inflammation by SAH. Conversely, 4-P-PDOT significantly increased cd86 mRNA levels and CD68 and iNOS protein expression, while decreasing cd206 and fizz mRNA levels and CD206 protein expression compared to the Mel+LPS group (Fig. 5E, F). Additionally, 4-P-PDOT increased il6 and tnfα mRNA levels and TLR4, p-P65, and p-IκB protein expression, while decreasing il10 mRNA levels compared to the Mel+LPS group (Fig. 5G). These data showed that 4-P-PDOT significantly reversed the changes induced by melatonin after LPS treatment.

### Melatonin protected the damaged colon barrier induced by LPS through m6A methylation modification of MTNR1B because of m6A methylation modification

**Figure 6:**
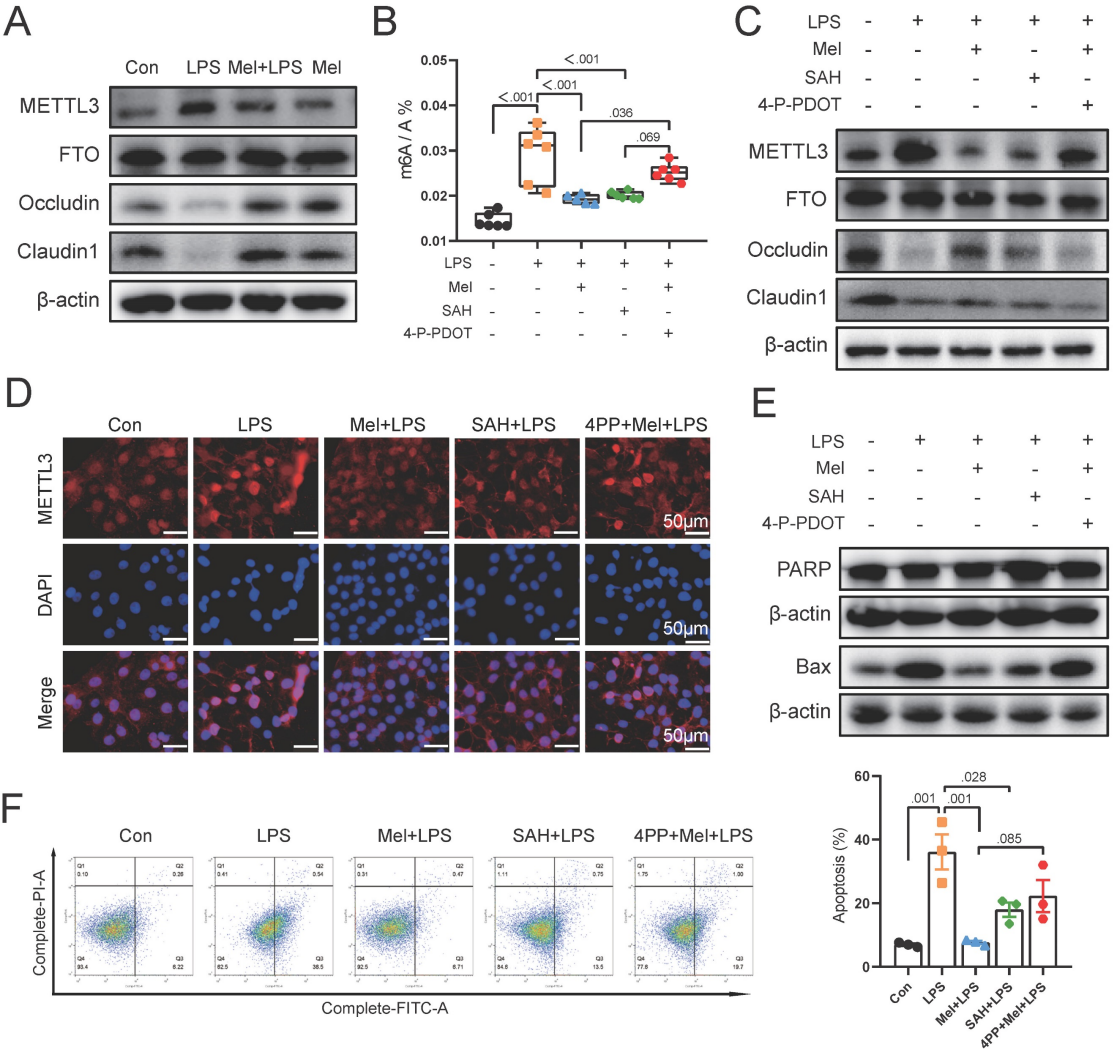
LPS damaged colon barrier and melatonin protected it through mtnr1b because of m6A methylation modification. (A) METTL3, FTO, Occludin and Claudin1 protein expression. (B) m6A level by LC-MS (n = 6). (C) METTL3, FTO, Occludin, and Claudin1 protein expression. (D) METTL3 staining. (E) PARP and BAX protein expression. (F) Statistics of apoptotic cells ratio which detected by flow cytometry (n = 3). Data are expressed as the mean ± SEM. One-way ANOVA. All p values are shown in the figures.

To further validate our findings in vivo, we selected the macrophage (RAW264.7) and colon cell (Caco2) co-culture model in vitro. Initially, we examined the protein expression of METTL3, FTO, Occludin, and Claudin1. The results were same as the results of colon (Fig. 6A). As a result of SAH and 4-P-PDOT treatment, we found that SAH inhibited m6A levels increased by LPS, whereas 4-P-PDOT increased m6A levels decreased by Mel+LPS treatment (Fig. 6B). The METTL3, Occludin and Claudin1 protein expression was also changed by SAH and 4-P-PDOT (Fig. 6C). Specifically, SAH inhibited METTL3 protein expression compared to the LPS group, whereas 4-P-PDOT reversed METTL3 protein expression compared to the Mel+LPS group. Notably, SAH reversed the decrease in Occludin and Claudin1 protein expression compared to the LPS group, while 4-P-PDOT inhibited it compared to the Mel+LPS group. METTL3 immunofluorescence staining revealed its nuclear expression, with the trend of METTL3 expression consistent with m6A levels (Fig. 6D). These results suggest an association between m6A methylation and colon barrier integrity. Finally, we assessed the expression of apoptosis proteins (PARP and BAX) in Caco2 cells using Western blotting. SAH inhibited apoptosis protein expression, whereas 4-P-PDOT promoted it (Fig. 6E). We further validated these findings using flow cytometry (Fig. 6F). In conclusion, our study suggests that LPS-induced colon inflammation is mediated by an increase in m6A methylation levels, which promotes the polarization of M1 macrophages and inflammation. Melatonin modulates m6A methylation levels via MTNR1B, promoting the polarization of M2 macrophages and exerting an anti-inflammatory effect.

### Melatonin via MTNR1B inhibited METTL3 expression that promotes macrophage transformation to M2 macrophages in mice

**Figure 7:**
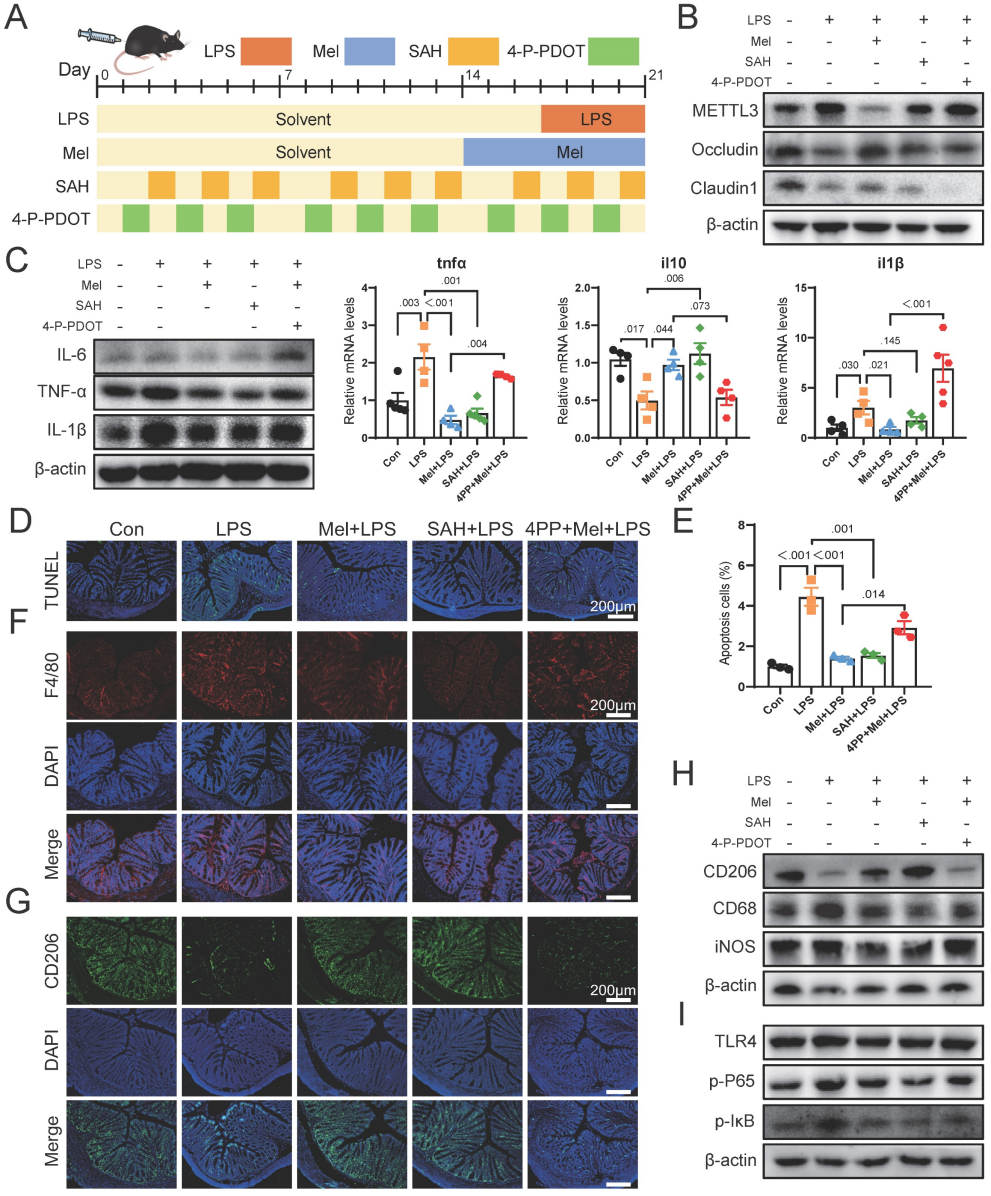
Melatonin through MTNR1B inhibited METTL3 expression that promotes M2 macrophages activation in mice. (A) Illustration of the experimental design. (B) METTL3, Occludin and Claudin1 protein expression. (C) IL-6, TNF-α and IL-1β protein expression and tnfα, il10 and il1β mRNA levels (n = 4). (D) TUNEL staining. (E) Apoptosis cells (n = 3). (E) tnfα, il10 and il1b mRNA levels (n = 4). (F) F4/80 staining. (G) CD206 staining. (H) CD206, CD68 and iNOS protein expression. (I) TLR4, p-P65, and p-IκB protein expression. Data are expressed as the mean ± SEM. One-way ANOVA. All p values are shown in the figures.

We then conducted further verification through mouse experiments (Fig. 7A). Firstly, we assessed METTL3 protein expression and observed that SAH inhibited LPS-induced overexpression of METTL3, while the effect of melatonin on reducing METTL3 expression was inhibited by 4-P-PDOT (Fig. 7B). The expression of Occludin and Claudin1 increased significantly after SAH treatment, while 4-P-PDOT significantly inhibited the influence of melatonin on tight junction protein expression. Additionally, the expression of Occludin and Claudin1 significantly increased after SAH treatment compared to the LPS group, while 4-P-PDOT significantly inhibited the influence of melatonin on tight junction protein expression. As shown in Fig. 7C, SAH significantly reduced the increase in LPS-induced pro-inflammatory factors (IL-6, TNF-α, and IL-1β) protein expression, whereas 4-P-PDOT significantly inhibited the action of melatonin (Fig. 7C). The il6, il10 and tnfα mRNA levels were also changed by SAH compared to the LPS group and 4-P-PDOT compared to the Mel+LPS group (Fig. 7C).

TUNEL staining showed that SAH effectively reduced cell apoptosis induced by LPS, while 4-P-PDOT significantly inhibited the anti-apoptotic effect of melatonin (Fig. 7D-E). Subsequent F4/80 and CD206 staining showed that SAH significantly reduced F4/80 positive cells and increased CD206 positive cells compared to the LPS group, whereas 4-P-PDOT inhibited the effect of melatonin, which improved LPS-induced effects (Fig. 7F-G). Western blot results of CD206, CD68 and iNOS further corroborated these findings (Fig. 7H). Finally, we assessed TLR4, p-P65, and p-IκB protein expression by Western blotting, revealing that SAH significantly inhibited compared to the LPS group, while 4-P-PDOT significantly increased TLR4, p-P65, and p-IκB protein expression compared to the Mel+LPS group (Fig. 7I). According to these results, melatonin suppresses excessive METTL3 expression via MTNR1B, enhances the activation of M2 macrophages, reduces cell apoptosis and improved colon inflammation induced by LPS.

### SAH and 4-P-PDOT changed the effects of LPS and melatonin on microflora

**Figure 8:**
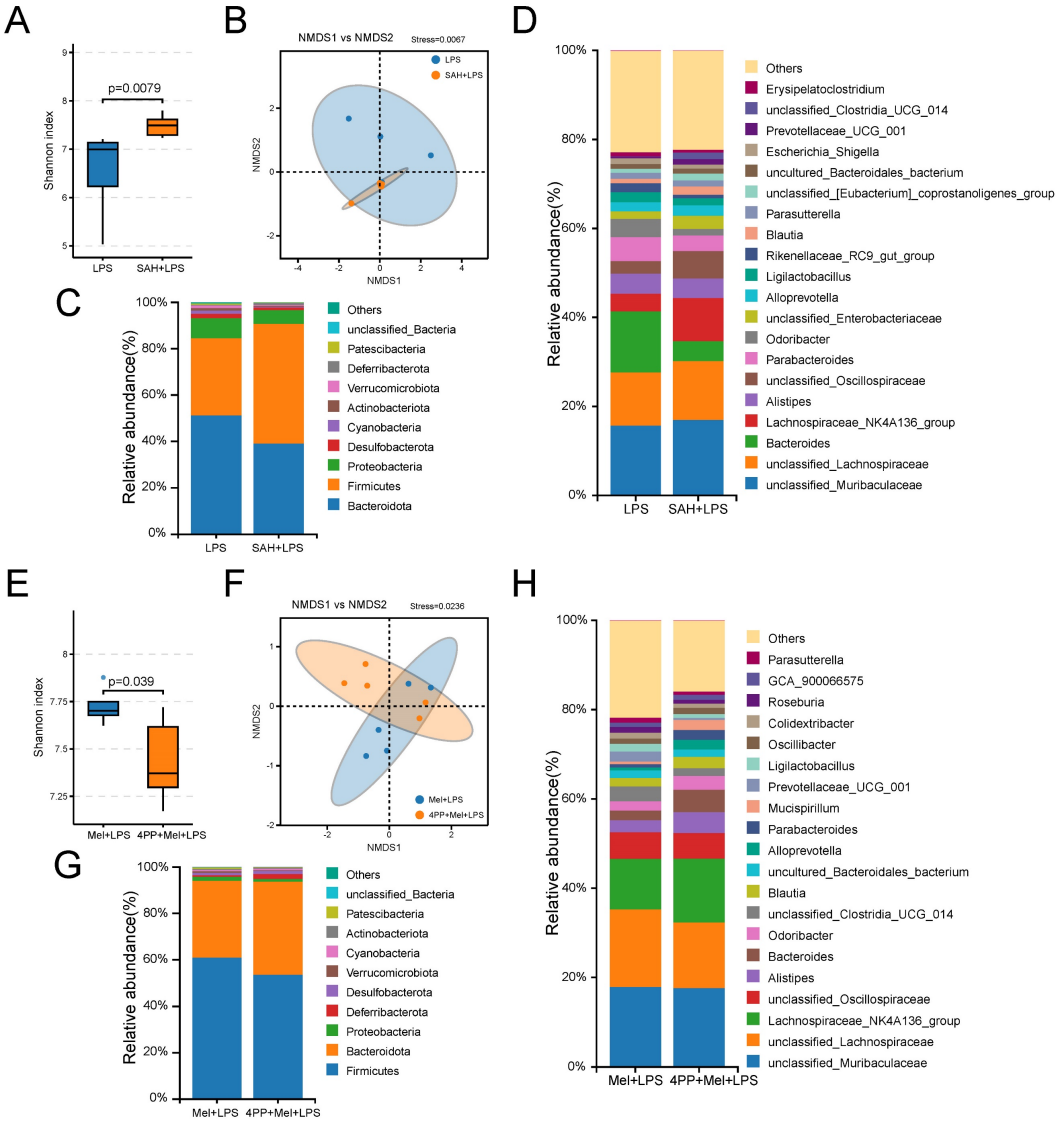
Composition of the colon microbiota after SAH and 4-P-PDOT treatment. (A) Shannon diversity indexes. (B) NMDS score plots based on the OTUs (n = 5). (C) Relative abundance of the top 10 genera at the phylum level. (D) Relative abundance of the top 20 genera at the genus level. (E) Shannon diversity indexes. (F) NMDS score plots based on the OTUs (n = 5). (G) Relative abundance of the top 10 genera at the phylum level. (H) Relative abundance of the top 20 genera at the genus level. All p values are shown in the figures.

Finally, we further examined the effects of SAH and 4-P-PDOT on the flora. It can be observed from the sequencing results that significant differences were noted in both alpha diversity (Shannon index) and beta diversity (NMDS), as well as in specific relative abundances following SAH treatment compared to the LPS group and 4-P-PDOT treatment compared to the Mel+LPS group (Fig. 8A, B, E, F). At the phylum levels, Firmicutes, Bacteroidota, and Proteobacteria were the main flora (Fig. 8C, G). At the genus levels, unclassified_Muribaculaceae, unclassified_Lachnospiraceae, Bacteroides, unclassified_Oscillospiraceae, and Parabacteroide were predominant in the SAH+LPS and LPS groups with significant alterations (Fig. 8D). Conversely, unclassified_Lachnospiraceae, Lachnospiraceae_NK4A136_group, unclassified_ Oscillospiraceae, Alistipes, and Bacteroides were predominant in the 4-P-PDOT+Mel+SAH and Mel+LPS groups with significant alterations compared to the Mel+LPS group (Fig. 8H).

## Discussion

Colon inflammation leads to intestinal barrier breakdown and disease. Improving the inflammatory response is the therapeutic goal of colon disease. Presently available treatments for colon inflammation involve anti-inflammatory agents and other inhibitors. However, these drugs have limited effectiveness. Furthermore, the gut microbiota influences on intestinal pathophysiology. In this study, we found that melatonin promotes M2 macrophage activation by inhibiting m6A hypermethylation through MTNR1B, maintaining colon flora homeostasis and improving colon inflammation.

Melatonin is a potent antioxidant known for its immunomodulatory and stress-relieving properties. Several studies have demonstrated that melatonin can regulate the activation of the immune system, thereby reducing chronic and acute inflammation 24,25. In this study, it was observed that melatonin can inhibit the abnormal expression of inflammatory factors induced by LPS. It can also increase the expression level of tight junctions, inhibit the apoptosis of colon cells, and maintain the stability of colon flora. Experimental findings revealed that LPS can lead to significant colon inflammatory infiltration and increased intestinal permeability. Additionally, it was found that LPS induced apoptosis and caused disturbance in colon flora, further exacerbating disease occurrence. These results are consistent with a previous study showing that melatonin alleviates disturbance in the colon microbiota induced by HFD 26. However, despite these findings, the mechanism of action of melatonin on inflammation remains unclear, further investigation is necessary to explore more evidence.

NF-κB plays a pivotal role in the activation of inflammatory genes mediated by TLR4 in macrophages. Our study revealed that melatonin inhibited the expression of TLR4/NF-κB proteins activated by LPS. These findings are consistent with a recent report 27, demonstrating that melatonin inhibits nuclear translocation of the NF-κB p50 subunit and its DNA-binding activity in *Prevotella* intermedia LPS-activated macrophages. Therefore, we propose that melatonin's inhibitory effect on TLR4-mediated inflammatory genes may be attributed, at least partially, to the suppression of NF-κB activation in macrophages. Macrophages exhibit either pro-inflammatory M1 (classically activated) or anti-inflammatory M2 (alternatively activated) macrophages based on different activation modes. M1 macrophages secrete pro-inflammatory cytokines such as TNF-α, IL-6, IL-1β and produce nitric oxide (NO), participating in inflammatory response and pathogen elimination to execute immune surveillance functions, whereas M2 macrophages generally produce anti-inflammatory factors such as IL-10 and TGF-β, promoting tissue repair and remodeling 28-30. Previous studies had proved that macrophages massively accumulate in the inflamed colon tissue and M1 macrophages were predominant in IBD patients 31. In this study, melatonin also promoted the differentiation of M2 macrophages.

A recent study revealed that m6A plays a key role in the regulation of inflammation. METTL3 has been identified as a pivotal methyltransferase essential to the performance of m6A modification 32. The absence or overexpression of METTL3 would surely change the total level of m6A methylation, directly impacting mRNA decay and translation, consequently leading to human diseases 32,33. Recent studies have reported that METTL3-mediated m6A is involved in the pyroptosis of diabetic retinopathy (DR) 34, and that METTL3 expression promotes macrophage apoptosis and inflammation in atherosclerosis (AS) and acute coronary syndrome (ACS) 35.

In this study, we initially observed an abnormal increase in METTL3 expression in the colon induced by LPS. This led us to believe that METTL3 plays a critical role in inflammatory responses. Furthermore, we found that Mettl3 expression was significantly up-regulated in patients with IBD according to clinical data. Based on these previous and current findings, the up-regulation of METTL3 in colon inflammation suggests potential involvement of m6A modification in the progression of colon inflammation. It has been reported that METTL3 may promote inflammatory response via the NF-κB signaling pathway, which exerts a significant impact on the occurrence and development of rheumatoid arthritis 36,37. Herein, SAH inhibited m6A methylation levels induced by LPS, and low levels of m6A methylation inhibited TLR4 expression and NF-κB phosphorylation in macrophages, suggesting the involvement of NF-κB signaling pathways in functions related to METTL3. In addition, we are surprised to find that inhibition of abnormal expression of m6A methylation levels also promoted the transformation of M1 macrophages induced by LPS to M2 macrophages. In contrast, METTL3 knockdown have been shown to have impaired colon barriers 38,39. To confirm the role of m6A in the colon inflammation, we examined the effects of SAH on Caco2 cells and RAW264.7 cells. Our findings indicated that SAH effectively reversed the LPS-induced inflammatory response in RAW264.7 cells and demonstrated protective effects on tight junction expression in Caco2 cells. Moreover, SAH inhibited LPS-induced apoptosis. In our study, these results indicated that METTL3 increased m6A methylation levels, activated NF-κB pathway of macrophages and promoted inflammation in colon.

Melatonin performs its biological functions mainly via these two important receptors, Mel1a (MTNR1A) and Mel1b (MTNR1B) 40. In our study, we found that melatonin workes through MTNR1B. Therefore, in vitro experiments, we further investigated whether the effects of melatonin are mediated by its receptors. The results showed that the inhibition of melatonin receptor by 4-P-PDOT affected the protective effect of melatonin. Surprisingly, we found that 4-P-PDOT increased m6A methylation levels, inhibited tight junction protein expression, and promoted apoptosis. These results suggested that melatonin regulates the methylation level of m6A through MTNR1B, regulates the inflammation and apoptosis of cells, to improve inflammation and protect the colon.

Our study provides evidence for the crucial role of m6A modification in maintaining colon epithelial cell homeostasis and elucidates the mechanism by which melatonin exerts its protective effect. Specifically, we found that LPS-induced upregulation of METTL3 and m6A modification in mice led to severe colon inflammation through activation of the TLR4/NF-κB-mediated inflammatory pathway in macrophages. Instead, melatonin protects the colon through MTNR1B to regulate the METTL3 protein and promote M2 macrophages. Our findings suggest that modulation of m6A methylation levels may be a promising therapeutic strategy for intervening in colon inflammation. Our study primarily analyzed the impact of m6A methylation on colon inflammation, without fully exploring the downstream effects of these methylation changes, which requires future research.

## Conclusion

The study demonstrated that melatonin could decrease the excessive expression of METTL3, thus mitigating the elevated m6A methylation levels induced by LPS. Specifically, LPS can mediate M1 macrophage activation and promote colon inflammation by promoting METTL3 protein overexpression. On the contrary, melatonin inhibits METTL3 expression through MTNR1B, promotes M2 macrophages activation, and alleviates colon inflammation.

## Figures and Tables

**Table 1:** Primers of target genes and reference genes.

Gene (mouse)	Forward primer (5′ - 3′)	Reverse primer (5′ - 3′)
mettl3	AAGGAGCCGGCTAAGAAGTC	TCACTGGCTTTCATGCACTC
mettl14	CTGAGAGTGCGGATAGCATTG	GAGCAGATGTATCATAGGAAGCC
mettl16	CTAGCAACCAAAGAGCAGGA	AGTCTTGACTGGGGAGTATGA
wtap	CCCGGGAGTACGAGCCC	TCATCATATTCCAGGCTTCCCA
kiaa1429	GCAGAGCAGCCTACAACGTGAC	CCAACATGCCAAGTATCAGGATCTC
fto	ACAAGATTAGATGCACCGCG	TGTCCATTTCCAGGATCCGG
alkbh5	ACAAGATTAGATGCACCGCG	TGTCCATTTCCAGGATCCGG
il1β	TCGCAGCAGCACATCAACAAGAG	TGCTCATGTCCTCATCCTGGAAGG
il6	ACTTCCATCCAGTTGCCTTCTTGG	TTAAGCCTCCGACTTGTGAAGTGG
tnfα	GGTGCCTATGTCTCAGCCTCTT	GCCATAGAACTGATGAGAGGGAG
il10	CTTACTGACTGGCATGAGGATCA	GCAGCTCTAGGAGCATGTGG
cd86	TGTTTCCGTGGAGACGCAAG	TTGAGCCTTTGTAAATGGGCA
cd206	CAGGTGTGGGCTCAGGTAGT	TGGCATGTCCTGGAATGAT
fizz	CAGAAGGCACAGCAGTCTTG	GGGTATTAGCTCCTGTCCCC
mtnr1a	TGTCAGCGAGCTGCTCAATG	GGTACACAGACAGGATGACCA
mtnr1b	GAACAGCTCAATCCCTAACTGC	ACGACTACTGTAGATAGCATGGG
